# HIV Disclosure and Prevention Advocacy Partially Mediate Advocacy Training Intervention Effect on Reduced Internalized HIV Stigma

**DOI:** 10.1007/s10461-026-05179-2

**Published:** 2026-05-23

**Authors:** Glenn J. Wagner, Laura M. Bogart, Stephen Okoboi, Violet Gwokyalya, Harold D. Green, David J. Klein, Susan Ninsiima, Joseph K. B. Matovu

**Affiliations:** 1 RAND, 1776 Main Street, Santa Monica, CA, USA; 2 Charles R. Drew University of Medicine and Science, Los Angeles, CA, USA; 3 Infectious Diseases Institute, Makerere University College of Health Sciences, Kampala, Uganda; 4 Makerere University School of Public Health, Kampala, Uganda; 5 University of Indiana Bloomington School of Public Health, Bloomington, IN, USA; 6 Faculty of Health Sciences, Busitema University, Mbale, Uganda

**Keywords:** Internalized HIV stigma, Intervention, Prevention advocacy, Disclosure, Mediators

## Abstract

Reduction of internalized HIV stigma is key to increasing HIV disclosure and engagement in peer advocacy for HIV prevention—and these latter processes may also be important for making further inroads into minimizing stigma. We examined mediators of the effect of a peer advocacy training intervention, Game Changers for HIV Prevention (GC-HIV), on reduced internalized HIV stigma among persons living with HIV (PLWH) in Uganda. A randomized controlled trial was conducted with 210 PLWH (105 in each of the intervention and usual care control arms) who were assessed at baseline and months 6, 12 and 18. Internalized HIV stigma was measured with the 6-item Internalized AIDS-Related Stigma Scale. A repeated measures linear regression model found an intervention effect on reduced internalized HIV stigma. Path analysis performed for each mediator separately revealed that this stigma reduction effect was partially mediated with significant indirect effects for increased HIV disclosure [beta (SE) = −0.05 (0.03); *p* = .046] and increased prevention advocacy [beta (SE) = −0.12 (0.05); *p* = .01], as well as a significant direct effect; inclusion of both these mediators in the model did not alter this result. HIV knowledge did not act as a mediator of the intervention effect on stigma. Findings support not only the value of the intervention for reducing internalized stigma, but also the benefits of HIV disclosure and encouraging others to protect against HIV for stigma reduction and self-acceptance for PLWH. Clinicaltrials.gov registration (NCT05098015), 2021–10–18.

## Introduction

Since the beginning of the pandemic, HIV has been the basis for targeted prejudice, discrimination and hostility towards persons living with HIV (PLWH) [1, 2]. Internalized HIV stigma is the acceptance of this societal devaluing, resulting in feelings of shame and low self-worth [3], and profound deleterious effects on the psychological and physical health of PLWH [1, 4–7]. Intervention research has identified social support, self-efficacy and empowerment to be among the mediators of effects on reduced internalized stigma [6, 8, 9]. Peer advocacy programs for HIV prevention, which also focus on empowerment and self-efficacy through skills building, knowledge and social support [10–13], have not only demonstrated effects on increased use of HIV protective behaviors [14–17], but also reduced internalized HIV stigma [10, 18]. These findings suggest that processes of peer advocacy among PLWH can help further the understanding of dynamics that serve to reduce internalized HIV stigma.

We developed the peer advocacy group training model, Game Changers for HIV Prevention (GC-HIV), to empower PLWH to act as change agents for HIV prevention through advocacy within their personal social networks. The conceptual model underpinning GC-HIV posits that increased advocacy among PLWH results from intervention content focused initially on reducing internalized HIV stigma [19]. Indeed, randomized controlled trials of GC-HIV showed that reduced internalized HIV stigma either fully or partially mediated effects of the intervention on increased advocacy for specific targeted HIV protective behaviors [19]. The intervention also had significant effects on reduced internalized HIV stigma, with greater reduction in stigma PLWH in the intervention group compared to those in the control group [19].

**Fig. 1:**
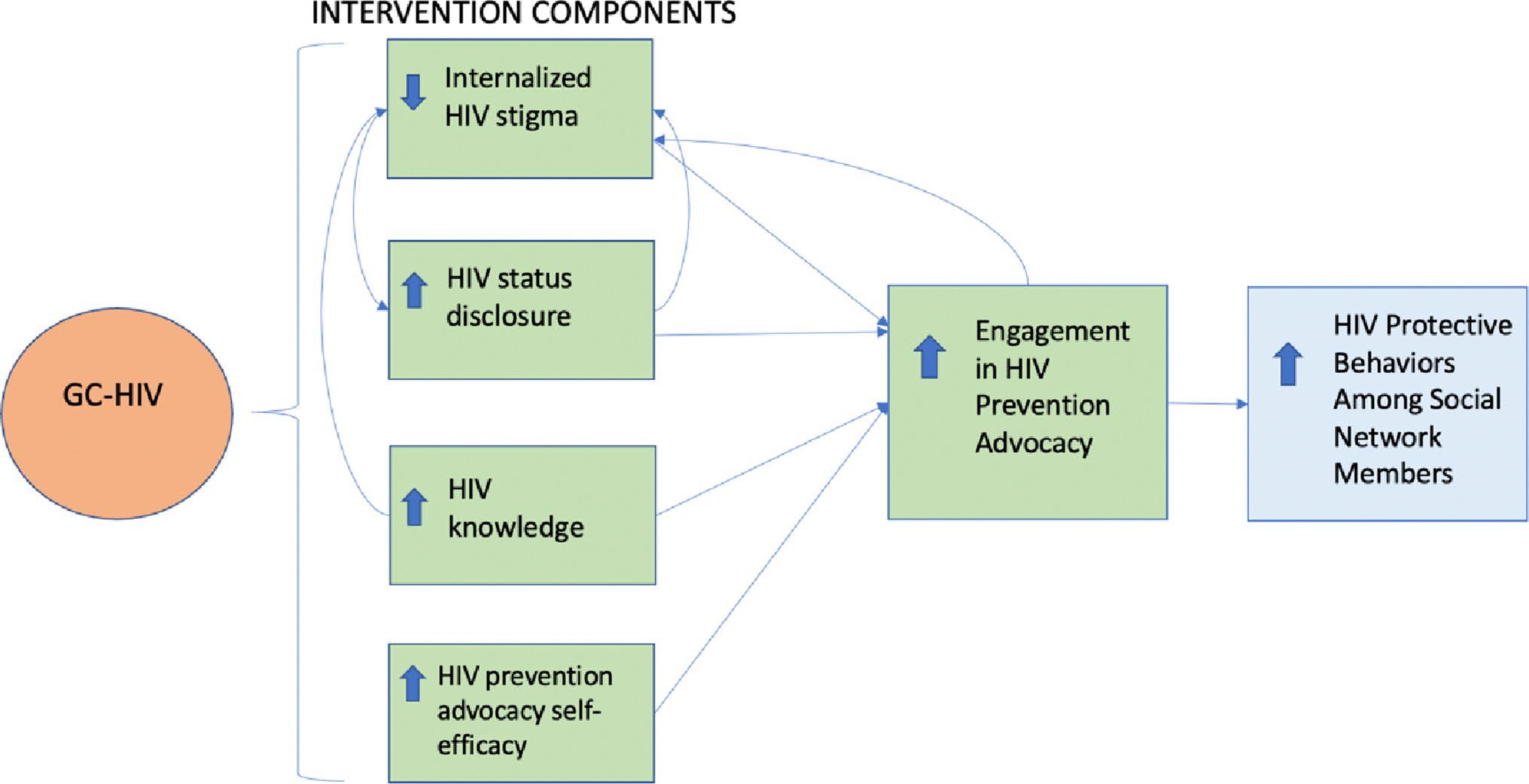
Conceptual framework for promotion of HIV prevention advocacy among persons living with HIV to affect HIV protective behaviors among social network members

Consistent with the Disclosure Processes Model [20], the Game Changers conceptual framework positions reduced internalized stigma as the starting point of a process that leads to increased HIV disclosure and experience sharing. The Disclosures Processes Model suggests that reduced internalized HIV stigma plays a critical role in HIV disclosure by lowering fear and increasing self-efficacy, which enable PLWH to feel comfortable and safe enough to disclose. When reduced internalized stigma is accompanied by increased HIV knowledge and advocacy self-efficacy, these processes are posited to increase engagement in advocacy. While these theoretical considerations support a causal direction in which stigma reduction leads to increased knowledge and advocacy through disclosure and experience sharing, these latter constructs in the theoretical pathway may also serve as mechanisms through which the intervention affects internalized stigma. Various related theoretical frameworks suggest the following processes that influence internalized HIV stigma: HIV disclosure reduces internalized stigma by shifting from avoidance to proactive action, and increasing access to social support (Disclosure Processes Model [20]; Social Support Theory [21, 22]); HIV knowledge reduces internalized stigma by challenging myths and building skills to address social biases (HIV Stigma Framework [23]; Social Ecological [24, 25]); and engagement in HIV prevention advocacy reduces stigma by building self-efficacy, community solidarity, and restoring social value (Social Ecological [24, 25]; Empowerment theory [26]). These theoretical considerations suggest that the associations between internalized HIV stigma and these related processes may be bidirectional, and that a circular feedback loop may be present (see Fig. 1). For example, reduced internalized stigma may lead to greater disclosure, resulting in increased access to social support, and in turn further reduction in internalized stigma.

With data from the GC-HIV randomized controlled trial, we examined whether the intervention effect on reduced internalized HIV stigma was mediated by increased HIV disclosure, increased HIV knowledge, and increased engagement in HIV prevention advocacy, with our hypotheses being that each of these constructs would at least partially mediate the intervention effect.

## Methods

### Study Design

The study was a parallel randomized controlled trial of the GC-HIV group peer advocacy intervention, conducted at The Infectious Diseases Institute in Kampala, Uganda. PLWH were enrolled as index participants and randomized (1:1 ratio) to receive either the intervention or a non-intervention usual care control group with stratification by sex. Each index participant was asked to recruit up to four social network members (alter participants) to whom they had disclosed their HIV status (from the up to 20 social network members they list in the assessment described below); however, only data from index participants were included in the analysis for this paper. After baseline, follow-up assessments were administered at months 6, 12 and 18. Participants received 30,000–70,000 Uganda shillings (~$8–20 USD), depending on the distance traveled, after completing each assessment, or attending each intervention session, to cover transportation costs. The study protocol was approved by ethical review boards at RAND (2021-N0155) and IDI (009–2021), and the Uganda National Council for Science and Technology (HS1896ES). Participants provided written informed consent. Further details of the study protocol are available in a prior publication [19]. The study has been registered with clinicaltrials.gov (NCT05098015, registered 2021–10–18).

### Participants

Recruitment took place between January 2022 and February 2023. Eligibility criteria for index participants included: (1) age ≥ 18 years, (2) in HIV care for at least one year (at which point they are more likely to have adjusted to their HIV diagnosis, disclosed their HIV status to several people, and thus, are more ready to engage in advocacy), (3) speak fluent Luganda or English, and (4) does not have a partner/spouse or household member already enrolled in the study as an index participant.

### Intervention

The intervention consisted of eight weekly group sessions, each of which lasted about 2 h. The session content focused on use of self-compassion and peer support to overcome internalized stigma, HIV disclosure decision making, HIV facts and myths, healthy positive living with HIV, and learning and practicing skills for HIV prevention advocacy (how to start and sustain conversations about HIV). In particular, the first session focused on reduction of internalized HIV stigma with the use of principles of compassion therapy [27] to encourage participants to share and acknowledge their experiences of hardship related to HIV, express compassion and empathy for themselves (and others), and gain self-acceptance as well as peer support and solidarity in recognition of how far they have come in overcoming these hardships. All sessions included sharing of experiences to build support; role playing to build skills and self-efficacy; setting personal goals regarding disclosure and advocacy; and take-home activities to reinforce new skills and generate personal experiences to be processed in the sessions. The sessions were conducted in Luganda or English (depending on the preference of the participants) by two trained Ugandan peer-facilitators using a structured manual. The facilitators were trained by the Ugandan and American senior investigators over three days. The Ugandan supervisor of the facilitators observed implementation of each session and provided feedback and further training as needed during weekly supervision.

### Measures

Survey and social network assessments were interviewer-administered using Network Canvas software [28] and conducted in Luganda or English, depending on the preference of the participant. All measures were translated from English to Luganda using a standard translation/backtranslation methodology in a pilot study [19]. Each assessment administered to index participants included a social network assessment in which the participant was asked to list 20 people in their social network (“alters”) with whom they interact most frequently. For each alter, the index participant was asked to provide information, as described below. All measures were administered at each assessment, unless otherwise noted.

#### Internalized HIV stigma

was measured using the 6-item Internalized AIDS-Related Stigma Scale [29]. Examples of items include “Being HIV-positive makes me feel like something is wrong with me” and “I feel guilty that I am HIV-positive”. Response options range from 1 ‘disagree strongly’ to 5 ‘agree strongly’. The mean item score was calculated; higher scores represent greater stigma.

#### HIV disclosure

was measured by calculating the percentage of alters to whom the participant reported having disclosed their HIV status.

#### HIV knowledge

was assessed with 13 statements related to the goals of HIV medication (e.g., “If a person with HIV infection does not take medication for HIV, their HIV viral load will increase”), drug resistance (e.g., “If you do not take HIV medication exactly as instructed, HIV in your body may become resistant to HIV medications”), adherence (e.g., “An HIV-positive individual does not need to take medication for HIV everyday if they do not have any symptoms”), HIV myths and misconceptions (e.g., “A person can get HIV through witchcraft of other supernatural means”), and HIV prevention (e.g., “Having an undetectable HIV viral load makes it very difficult for you to transmit the virus to someone else”). Some items were developed by the study team, while others were derived from the Patient’s HIV Knowledge Questionnaire [30]. Response options consisted of ‘true,’ ‘false,’ ‘don’t know,’ and ‘not sure,’ and a sum of correct responses was calculated.

#### HIV prevention advocacy.

For each alter named in the social network assessment, index participants were asked in separate yes/no questions if they had talked with the alter in the past three months about (1) condom use, (2) HIV testing or (3) PrEP use if the alter was not living with HIV, and if the alter was living with HIV—(4) engagement in HIV care and (5) use of ART. For analysis, a binary derived variable was created for each alter to indicate whether the index participant had engaged in advocacy for any of the five protective behaviors with the alter; the percentage of alters targeted with any advocacy was calculated.

#### Background demographic and medical characteristics.

Index participants reported their age (in years), sex (male, female), highest level of education, relationship status, and length of time (in years) since HIV diagnosis and in HIV care. Chart abstraction was used to collect data on CD4 count, viral load and use of ART.

### Data Analysis

Bivariate statistics (independent 2-tailed t-tests; chi-square tests) were used to compare background characteristics of index participants between those in the intervention and controls groups. To test intervention effects on the three potential mediators (HIV disclosure, HIV knowledge, HIV prevention advocacy) measured at month 6, we compared these measures between the intervention and control arms using linear regression analysis and controlling for index age, sex, secondary education, relationship status, and the baseline measures of internalized HIV stigma and the mediator. Separate models were conducted for each mediator.

Prior published analyses revealed a significant intervention effect on reduced internalized HIV stigma among index participants [19]. To examine mediators of this effect, for each potential mediator, a path analysis was conducted with three functional relationships specified. The first path specified that the intervention, expressed as an indicator of study arm, affected the primary dependent variable (internalized HIV stigma averaged across all follow-up assessments) as well as the mediator measured at month 6. The second path specified that the mediator measured at month 6 affected the primary dependent variable. The third path specified that index demographic characteristics (age, secondary education, sex, and relationship status), the mediator at baseline, and the baseline measure of the dependent variable affected the primary dependent variable at follow-up as well as the mediator at month 6. These models were run for each mediator individually, in separate analyses. However, if multiple mediators were found to partially mediate the intervention effect on the primary dependent variable, a subsequent model was run that included those mediators (measured at baseline and month 6) to assess whether jointly these mediators would fully mediate the intervention effect on advocacy. All path models were just-identified (df = 0); therefore, global fit indices are necessarily saturated and do not provide a test of model specification. R^2^ values for endogenous variables are reported as indicators of explanatory adequacy. Regression coefficients for all paths as well as for direct and indirect effects were estimated with SAS v9.4 Proc Calis. Given that the mediators were variables measured at month 6, the overall analytic sample was restricted to index participants who completed the month 6 assessment.

## Results

### Sample Characteristics

Two hundred and ten index participants (105 in each of the intervention and control arms) enrolled in the study, of whom 208 completed the month 6 assessment and comprised the analytic sample; of these 208 participants, 202 (97.1%) completed month 12 and 204 (98.1%) completed month 18. These 208 index participants named 3,185 alters at baseline (mean = 15.3 alters per index participant in both intervention and control arms). Table 1 shows background characteristics of index participants, in the whole sample as well as within the control and intervention arms; there were no statistically significant differences between arms on any of these characteristics. Mean age was near 40 years, nearly a third were male, the majority had at least some secondary education and two-thirds were married or in a committed relationship. Most had been HIV diagnosed [mean (SD) = 12.8 (6.0) years] and in HIV care [mean (SD) = 11.0 (5.6) years] for many years; mean CD4 cell count was high [mean (SD = 527 (291)] and a high majority had undetectable viral load (90.9%).

### Intervention Effects on Internalized HIV Stigma and the Potential Mediators

In a prior paper, we documented a significant intervention effect on reduced internalized HIV stigma among index participants [19]. A similar result is shown in the first model (no mediator) listed in Table 2. Among index participants in the intervention group, mean stigma decreased from 2.4 at baseline to 1.6 at month 6, where it remained through month 18; in the control group, mean stigma was 2.3 at baseline and it remained stable throughout follow-up (mean 2.0–2.1.0.1). Examining intervention effects on each of the potential mediators revealed that the intervention also had a significant effect on each of the mediators at month 6 after controlling for the baseline measures of the mediator, internalized HIV stigma, and index background characteristics (age, secondary education, sex, and relationship status): HIV disclosure [beta (SE) = 7.02 (2.89); *p* =.02], HIV knowledge [beta (SE) = 0.48 (0.16); *p* =.002], and HIV prevention advocacy [beta (SE) = 25.10 (3.51); *p* <.001]. The three potential mediators were all significantly correlated with each other: HIV prevention advocacy was positively correlated with HIV knowledge (*r* =.35; *p* < .001) and HIV disclosure (*r* =.28; *p* < .001), and HIV knowledge was positively correlated with HIV disclosure (*r* =.15; *p* = .03).

### Mediation of the Intervention Effect on Internalized HIV Stigma

Table 2 present the results of the path analysis examining mediation of the intervention effect on reduced internalized HIV stigma, with each mediator examined separately. These analyses revealed that both increased HIV disclosure [beta (SE) = −0.01 (0.002); *p* =.001] and increased HIV prevention advocacy [beta (SE) = −0.005 (0.002); *p* =.004] at month 6 were significantly associated with reduced stigma across the 18-month follow-up. Further, each partially mediated the intervention effect on reduced stigma, with a significant indirect effect for both disclosure [beta (SE) = −0.05 (0.03); *p* =.046] and prevention advocacy [beta (SE) = −0.12 (0.05); *p* =.01], but with the direct effects of the intervention also remaining significant. Increased HIV knowledge was marginally associated with reduced stigma and did not act as a mediator.

With the above results showing that both increased HIV disclosure and increased prevention advocacy had each partially mediated the intervention effects on reduced internalized HIV stigma, and the modest correlation between the two constructs, both mediators were then included in the analysis to assess whether their roles as joint mediators may lead to full mediation of the intervention effect on reduced stigma. With both mediators in the model, the intervention effect on reduced stigma remained partially mediated (see Table 2), with both a significant indirect [beta (SE) = −0.14 (0.05); *p* =.004] and direct effect.

## Discussion

Game Changers for HIV Prevention (GC-HIV) is a peer advocacy training program that has been shown to increase HIV prevention advocacy among persons living with HIV (PLWH), in addition to effects on the components of the intervention, including reduced internalized HIV stigma [19]. The analysis reported here sought to better understand the mechanisms by which the intervention acts on internalized stigma. Despite internalized HIV stigma preceding HIV disclosure and HIV prevention advocacy in the theoretical pathway and structural focus of the intervention, our findings revealed that increased HIV disclosure and prevention advocacy, both of which were also targeted components that had been impacted by the intervention [19], partially mediated the intervention effects on reduced internalized stigma.

Internalized HIV stigma was generally low in this sample of PLWH, which was likely a result of the eligibility criteria: the study purposively selected PLWH who had been in HIV care for at least a year, with the assumption that this would render a sample with stable health, adequate adjustment to HIV diagnosis, and a readiness to use their experience to help others. The nature of the intervention self-selected for PLWH with a motivation to protect others from HIV and to learn how to engage in more effective HIV prevention advocacy. These selection biases should be kept in mind when considering for whom our study findings will have greater application. A sample that was more representative of the general population of PLWH in Uganda, or those newly diagnosed or not yet engaged in HIV care, may have higher levels of internalized HIV stigma and thus revealed even stronger effects of the intervention on stigma reduction; nonetheless, the level of internalized stigma in our sample was high enough to detect a significant reduction among those in the intervention arm, compared to the control group. The intervention’s first session used compassion therapy principles to encourage participants to recognize any hardship they had experienced from HIV, and to show themselves (and others) compassion and empathy for how far they had come in overcoming this hardship. Our qualitative findings from recipients of the intervention reaffirmed how the process of sharing personal experiences with hardship (e.g., experienced HIV-related stigma and discrimination), as well as stories of HIV disclosure and receipt of social support, resulted in a sense of solidarity and the “walls of stigma coming down” [19].

While GC-HIV was designed to first increase self-acceptance and reduce internalized HIV stigma, such that this would promote increased comfort with HIV disclosure, personal sharing of HIV experiences and engagement in prevention advocacy, our analyses showed that these latter processes served as partial mediators of the intervention effect on reduced stigma. These findings are consistent with theories of stigma reduction [20, 25, 26] and support our hypotheses that feedback loops may exist between processes of internalized stigma reduction and both increased HIV disclosure and increased prevention advocacy. Reduced stigma enables someone to have the courage to disclose, and as most disclosure events in the study setting result in positive, supportive responses [31], the disclosure process may further reduce internalized stigma and promote further disclosure. Similarly, reduced internalized stigma promotes comfort with experience sharing and encouraging HIV protective behaviors through advocacy, which may increase feelings of agency and purpose and result in others expressing gratitude and support.

While our partial mediation results suggest the potential for the presence of feedback loops between the processes of stigma, disclosure and advocacy, the temporal positions of our assessments and observed changes did not allow us to establish the presence of these feedback loops. Most of the intervention effect on reduced stigma took place between the baseline and month 6 assessment. To assess the feedback loop, multiple assessments would need to be added to the first six months of the study to enable temporal ordered measurement of stigma, followed by disclosure and/or advocacy (once some but not all reduction in stigma has taken place), and then stigma again, prior to the intervention’s effects being fully actualized. It is also possible that these processes occur nearly contemporaneously, making it practically infeasible to time the assessments to test the causal sequence. Future research may consider using ecological momentary assessments for individuals to collect real-time data on their feelings and thoughts as events such as HIV disclosure and advocacy occur [32].

In conclusion, the GC-HIV peer advocacy training intervention significantly reduced internalized HIV stigma among PLWH, and this effect was partially mediated by its increase of HIV disclosure and engagement in HIV prevention advocacy among social network members. These findings support not only the value of the intervention for reducing internalized stigma, but also the benefits of HIV disclosure and encouraging others to protect against HIV for stigma reduction and self-acceptance for PLWH. Just as HIV disclosure and prevention advocacy are behaviors that provide PLWH with a vital role in the fight against HIV by protecting others from HIV acquisition [33, 34], they are also central to breaking down internalized stigma and rebuilding self-worth and esteem for PLWH. Research is needed to further examine the potential feedback loop between HIV stigma reduction, disclosure and prevention advocacy, thereby enabling support programs to optimize these processes for the health and well-being of PLWH and those in their social networks and communities.

## Figures and Tables

**Table 1:** Baseline characteristics of index participants in the whole sample and within each study arm

Number of index participants	Total	Control	Intervention	t_df_ or χ^2^	*p*

	208	103	105	--	--

Age	40.0 (10.6)	39.3 (10.5)	40.7 (10.7)	t_206_ = 0.92	0.36
Male	63 (30.3%)	32 (31.1%)	31 (29.5%)	χ^2^ = 0.06	0.81
Any secondary education	124 (59.6%)	67 (65.1%)	57 (54.3%)	χ^2^ = 2.50	0.11
Married or in committed relationship	138 (66.4%)	68 (66.0%)	70 (66.7%)	χ^2^ = 0.01	0.92
Years since HIV diagnosis^a^	12.8 (6.0)	13.0 (5.6)	12.5 (6.3)	t_183_ = 0.54	0.59
Years in HIV care^b^	11.0 (5.6)	11.2 (5.2)	10.8 (6.1)	t_203_ = 0.52	0.60
CD4 count (cell/mm^3^)^c^	527 (291)	528 (326)	527 (254)	t_187_ = 0.02	0.98
Last HIV viral load was undetectable^d^	150 (90.9%)	71 (91.0%)	79 (90.8%)	χ^2^ = <0.01	0.96
Internalized HIV stigma	2.3 (1.1)	2.3 (0.9)	2.4 (1.0)	t_199_ = 0.39	0.70
HIV knowledge	10.6 (1.3)	10.5 (1.3)	10.7 (1.4)	t_206_ = 1.28	0.20
HIV prevention advocacy (% alters targeted with any advocacy)	30.6 (26.5)	31.8 (27.8)	29.5 (25.4)	t_206_ = 0.63	0.53
HIV disclosure (% alters disclosed HIV to)	51.2 (32.2)	48.5 (31.1)	53.9 (33.2)	t_206_ = 1.21	0.23

The p-values are based on t-test for continuous characteristics and chi-square tests for binary characteristics

a *N* = 185

b *N* = 205

c *N* = 202

d *N* = 165

**Table 2:** Regression models examining change (from baseline to month 6) in HIV disclosure, HIV knowledge, and HIV prevention advocacy as mediators of the intervention effect on mean internalized HIV stigma across all 18 months of follow-up

	No mediator		HIV Disclosure		HIV Knowledge		HIV Prevention Advocacy		Combined Model	
					
	Beta (SE)	*p*	Beta (SE)	*p*	Beta (SE)	*p*	Beta (SE)	*p*	Beta (SE)	*p*

Intervention (Direct effect)	−0.45 (0.09)	<0.001	−0.39 (0.09)	<0.001	−0.41 (0.09)	<0.001	−0.33 (0.10)	<0.001	−0.30 (0.09)	0.002
Internalized HIV stigma (BL)	0.37 (0.04)	<0.001	0.34 (0.04)	<0.001	0.36 (0.04)	<0.001	0.35 (0.04)	<0.001	0.33 (0.04)	<0.001
HIV disclosure (BL)	--		0.001 (0.002)	0.66	--		--		0.0003 (0.002)	0.87
HIV disclosure (M6)	--		−0.01 (0.002)	0.001	--		--		−0.01 (0.002)	0.002
HIV knowledge (BL)	--		--		0.01 (0.03)	0.77	--		--	
HIV knowledge (M6)	--		--		−0.07 (0.04)	0.09	--		--	
HIV prevention advocacy (BL)	--		--		--		0.001 (0.002)	0.43	0.002 (0.002)	0.27
HIV prevention advocacy (M6)	--		--		--		−0.005 (0.002)	0.004	−0.004 (0.002)	0.02
Indirect effect	--		−0.05 (0.03)	0.046	−0.03 (0.02)	0.13	−0.12 (0.05)	0.01	−0.14 (0.05)	0.004
R^2^ for outcome	0.3981		0.4398		0.4068		0.4215		0.4516	
R^2^ for mediator	--		0.4574		0.1830		0.2497		0.4575 (disclosure); 0.2516 (advocacy)	

All models included the following covariates: age, secondary education, sex and relationship status of index participant

## Data Availability

De-identified dataset and statistical code are available to researchers upon submission of the proposal and review by the study team.
